# An Interpretable Machine Learning Approach to Predict Sensory Processing Sensitivity Trait in Nursing Students

**DOI:** 10.3390/ejihpe14040059

**Published:** 2024-04-02

**Authors:** Alicia Ponce-Valencia, Diana Jiménez-Rodríguez, Juan José Hernández Morante, Carlos Martínez Cortés, Horacio Pérez-Sánchez, Paloma Echevarría Pérez

**Affiliations:** 1 Faculty of Nursing, Universidad Católica de Murcia, Campus de Guadalupe, 30107 Murcia, Spain; aponce@ucam.edu (A.P.-V.); pechevarria@ucam.edu (P.E.P.); 2 Faculty of Health Sciences, Universidad de Almería, Carretera Sacramento s/n, 04120 Almería, Spain; djr239@ual.es; 3 Structural Bioinformatics and High-Performance Computing (BIO-HPC) Research Group, Universidad Católica de Murcia, Campus de Guadalupe, 30107 Murcia, Spain; cmartinez1@ucam.edu (C.M.C.); hperez@ucam.edu (H.P.-S.)

**Keywords:** sensory processing sensitivity, emotional intelligence, conflict resolution, communication skills, nursing, interpretable machine learning

## Abstract

Sensory processing sensitivity (SPS) is a personality trait that makes certain individuals excessively sensitive to stimuli. People carrying this trait are defined as Highly Sensitive People (HSP). The SPS trait is notably prevalent among nursing students and nurse staff. Although there are HSP diagnostic tools, there is little information about early detection. Therefore, the aim of this work was to develop a prediction model to identify HSP and provide an individualized nursing assessment. A total of 672 nursing students completed all the evaluations. In addition to the HSP diagnosis, emotional intelligence, communication skills, and conflict styles were evaluated. An interpretable machine learning model was trained to predict the SPS trait. We observed a 33% prevalence of HSP, which was higher in women and people with previous health training. HSP were characterized by greater emotional repair (*p* = 0.033), empathy (*p* = 0.030), respect (*p* = 0.038), and global communication skills (*p* = 0.036). Overall, sex and emotional intelligence dimensions are important to detect this trait, although personal characteristics should be considered. The present individualized prediction model could help to predict the presence of the SPS trait in nursing students, which may be useful in conducting intervention strategies to avoid the negative consequences and reinforce the positive ones of this trait.

## 1. Introduction

Sensory processing sensitivity (SPS) can be defined as a personality trait that reflects individual characteristics of sensitivity to internal and external stimuli [1]. People carrying this trait are known as Highly Sensitive People (HSP) and are characterized by high emotional and empathic responsiveness and deeper information processing capabilities [2]. It is important to note that being highly sensitive is considered a neutral psychological trait and not a disorder. While HSP have several strengths, such as being more aware of their environment or having better information processing, it may also come with challenges, such as a higher likelihood of experiencing stress or anxiety in certain situations [3].

Aron describes high sensitivity as a dual-faceted attribute encompassing both favorable and unfavorable dimensions [4]. HSP are believed to be particularly sensitive to sensory stimulation, easily excitable, and particularly attentive to aesthetic impressions [5]. Despite the potential desirability of these characteristics, on the contrary, HSP struggle to find their place in societies whose members, for the most part, do not share these characteristics. For instance, HSP are distinguished by great emotional reactivity and empathy, which could be described as higher emotional intelligence, as well as the ability to process information more deeply [6], which makes them more vulnerable to external influences, easily influenced, and susceptible to sudden hyperactivity [7].

Initially, the first investigations viewed sensitivity as a vulnerability [8]. However, recent studies have revealed adaptive characteristics of individuals with elevated levels of SPS, such as more positive emotions in supportive environments [9,10]. Recent studies also demonstrate that individuals with elevated levels of SPS have a heightened ability to respond to both positive and negative experiences [11]. This special sensitivity towards the environment has implications for health, education, and work. In fact, authors such as Costa-López et al. have hypothesized that HSP would have a decrease in communication skills [12], which are so relevant in the interaction between patients and healthcare professionals. This is especially important considering that the prevalence of SPS in the general population is higher than expected, placing this figure around 30% [13,14]. Moreover, it has also been described that SPS is a crucial factor that not only affects happiness or quality of life but also functional or physiological levels [1,12].

Aron et al. believe that SPS is a key factor that affects not only health or quality of life but also at a functional or physiological level [4]. Moreover, SPS is biologically associated with heightened awareness and the ability to respond to environmental and social stimuli [4]. These people are characterized by being good observers and highly creative. However, they are introverts and can easily suffer from important levels of stress. As a whole, although it has not yet been studied in depth, HSP could have difficulties with conflict resolution both in work, social, or educational environments.

During their college years, nursing students spend much time in classrooms, clinical simulation rooms, and academic clinics, where they are exposed to a variety of life experiences that are emotionally challenging and can affect their future development and well-being. Some studies have hypothesized that stress is pervasive in all aspects of undergraduate nursing education [11,15] and that SPS may negatively respond to these environments and develop stress-related problems [16]. This period of study is also characterized by increased vulnerability to various mental health challenges [17], and therefore, the educational context can have an important influence on students’ personal and professional development. In this regard, positive educational environments may reinforce the positive features of SPS. However, the impact of environmental factors is subjected to individual variability, wherein a context considered positive by some may manifest as adverse for others.

The identification of the SPS trait in nursing students is of great relevance, particularly from an individualized perspective, which may help to conduct the necessary interventions to promote the positive aspects of this trait. Within this context, machine learning (ML) emerges as a field of study in the branch of algorithm evolution that gives computers the ability to learn without being explicitly programmed and emulate human intelligence learning from their data environment [18]. ML algorithms allow for the development of predictive models at the individual level; given the complexity of the SPS trait and its correlation with specific emotion regulation difficulties, relying solely on classical regression models, which necessitate the formulation of a priori hypotheses, may limit the exploration of these connections. On the other hand, ML techniques investigate probabilistic relationships between variables and employ repeated cross-validation methods to ascertain the reliability of findings [19]. Supervised machine learning entails the development of a statistical model based on a set of example or training data, which aid in pattern recognition for the future modeling of new datasets. Indeed, the application of machine learning in psychology and psychiatry research has seen a significant increase, and ML techniques have been employed for detecting depression, anxiety, and apathy [20], posttraumatic stress disorder [21], and other psychiatric conditions (schizophrenia, anorexia, substance abuse) [22]. However, to the best of our knowledge, no earlier study has been conducted with this methodology within the context of HSP. In this line, several machine learning algorithms were conducted to analyze the particularities of SPS through their interaction with emotion regulation, social skills, and conflict resolution styles. Therefore, the objective of the present study was to evaluate the presence of the SPS trait in nursing students and to evaluate the possible differences in emotional intelligence dimensions, communication skills, and conflict resolution styles. All of this is in order to try to develop a predictive model that allows us to detect HSP early, which, in turn, would help targeted interventions aimed at reinforcing the advantageous characteristics associated with this trait.

## 2. Materials and Methods

### 2.1. Study Design and Participants

The present cross-sectional study was conducted in a university setting. Data collection took place during 2022. For the development of the present work, we initially collected data from 1190 nursing students, of which only 672 completed all evaluations, and therefore, were considered the study sample.

The form was submitted in digital Google Form format. A single form including all determinations was developed. A QR code was made to facilitate access to the questionnaire. Each student filled out the form from their electronic devices. A researcher was present to make the pertinent instructions for carrying out the tests. Previously, an explanatory presentation was designed on how to fill out the forms.

The study was approved by the Ethics Committee of the University (#CE061902), respecting the agreements of the Declaration of Helsinki of 1964. The participants were informed of the characteristics of the study in addition to the purpose of the data obtained. By completing the questionnaire, they gave their consent. The data collection process was conducted during class time and the decision to take part was free and voluntary, without a compensation agreement and without disadvantages for students who chose not to take part.

### 2.2. Data Collection Instruments

The following instruments were used to collect the data:

Custom sociodemographic variables questionnaire: A specific survey was designed for this study. The questionnaire was composed of six items: sex, age, marital status, cohabitation group, and self-perception of the quality of relationships with family, couples, and social mates.

Reduced Scale for Highly Sensitive People (r-HSP): The validated diagnostic test for finding individuals with the sensory processing sensitivity (SPS) trait is the 27-item Highly Sensitive Persons scale (HSP scale), developed by Aron and Aron [1]. This instrument is extensively used to assess the environmental sensitivity of both students and adults. Originally, the HSP scale, consisting of twenty-seven items, was developed by Aron and Aron in 1997. The present work employed the reduced version (r-HSP), including sixteen items with values ranging from 0 to 16; the r-HSP scale measures sensitivity, with a higher score showing greater sensitivity. The reliability of the Spanish version of the r-HSP scale was assessed through Cronbach’s α, yielding an acceptable value of α = 0.702 [23].

Test of perceived emotional intelligence (TMMS-24): The Trait Meta Mood Scale 24 is a measurement instrument based on the emotional meta-knowledge of the subjects, based on which three dimensions of emotional intelligence, attention, clarity, and emotional repair, are evaluated. Originally, it had forty-eight items, with Likert-type responses. The TMMS-24 is a reduced version adapted to Spanish, developed by Fernández Berrocal et al. [24,25], which keeps the same format as the original TMMS [25], with good psychometric qualities for the Spanish-speaking population. Through twenty-four items, it evaluates three factors or dimensions (eight items per factor): attention to feelings, emotional clarity, and repair of emotions. Attention to feelings is the degree to which people believe they pay attention to their emotions and feelings (i.e., “I think about my mood constantly”); emotional clarity refers to how people believe they perceive their emotions (“I frequently make mistakes with my feelings”); and finally, emotional repair refers to the subject’s belief in their ability to interrupt and regulate negative emotional states and prolong positive ones (i.e., “Although sometimes I feel sad, I usually have an optimistic outlook.”). The internal reliability of the original instrument was 0.95 (95%). Likewise, for each of the dimensions, the Cronbach’s alpha coefficient measurements obtained were greater than 85%.

Scale on communication skills in health professionals (EHC-PS): This scale is composed of eighteen items, with a Likert-type response scale. This scale has been shown to have adequate psychometric properties. The reliability analysis showed (internal consistency with Cronbach’s α in all its dimensions between 0.65 and 0.78). The four dimensions that make up the scale are informative communication, composed of 6 items, which reflects the way that health professionals obtain and provide information in the clinical relationship that they establish with patients; empathy, composed of 5 items, which reflects the ability of health professionals to understand the patients’ feelings, as well as the empathic attitude, including active listening and empathic response. The factor respect, composed of three items, evaluates the respect that health professionals show in the clinical relationship they establish with patients. Finally, the social skills factor reflects the ability of health professionals to be assertive or socially skilled in the clinical relationship they set up with patients [26].

Thomas–Kilmann’s Conflict Style Assessment: The Thomas–Kilmann Conflict Survey Instrument (TKI) is a forced-choice measure comprised of thirty statement pairs, each illustrating one of the five conflict modes. Respondents are required to select one statement from each pair that best represents their approach to conflict situations. Each conflict mode is presented 12 times, allowing for a maximum score of 12 for each mode. Scores are then categorized as either HIGH or LOW for each conflict mode based on whether they fall within the top or bottom 25th percentile, respectively.

Participants were instructed to complete the TKI instrument considering their responses in a work context, rather than a home setting. The Thomas–Kilmann Conflict Survey Instrument assesses five conflict resolution strategies: avoidance, accommodation, competition, compromise, and collaboration. Widely recognized as one of the premier tools for identifying conflict management styles, the TKI has demonstrated reliability coefficients ranging from 0.61 to 0.68 in test–retest analyses and between 0.43 and 0.71 with Cronbach’s alpha [27].

### 2.3. Statistical Analysis

Basic descriptive statistics of frequency (percentage) and dispersion (mean and standard deviation) were analyzed. Taking into account the high sample size, normality was studied according to the Kruskal–Wallis test. Furthermore, to analyze the relationship between qualitative variables (nominal or ordinal), such as the relationship between sex and the diagnosis (positive or negative) of HSP, the chi-square test was used. When the mean values of two independent groups were analyzed, the *t* test was analyzed. The effect size in this case was measured with Cohen’s d statistic. When more than two groups were evaluated, a one-way ANOVA was used, where the eta-squared value was used as a measure of the effect size. To develop the HSP predictive model, a multivariable logistic regression procedure was used, where variables were included according to the stepwise procedure. Only those variables with any significant association with the diagnosis of HSP were included in the first model. All statistical analyses were conducted with SPSS 27.0 software. A value of statistical significance was established for a value of *p* < 0.050.

### 2.4. Machine Learning Procedure

Machine learning (ML) is a branch of artificial intelligence that focuses on the development of algorithms and models that can learn and make predictions or decisions based on data, without being explicitly programmed. In the context of our study, we employed ML techniques to develop predictive models for identifying the presence of the SPS trait among nursing students. By leveraging the power of ML, we aimed to uncover complex patterns and relationships in the data that may not be apparent through traditional statistical methods.

The first step of the machine learning (ML) procedure was a meticulous preprocessing to ensure the integrity and accuracy of the analysis, which involved outlier removal and data curation. Next, we addressed the issue of low variance in the dataset. Variables that showed less than 5% variance were removed, because variables with minimal variance contribute little to no meaningful information and can impede the performance of predictive models. In addition, those variables exhibiting high correlation were also removed from the final dataset. Specifically, variables with more than 90% correlation were eliminated. This action helps to reduce multicollinearity, ensuring that our models are not biased or overfitted due to highly correlated predictors. In addition, we applied one-hot encoding to several categorical variables to better capture their nuances in our analysis. These variables included ‘Academic Year’, which is the academic period of the study; ‘Previous Health Education’, indicating the level of health education received prior to the study; ‘Perception of Relationships with Relatives’, ‘Perception of Relationships with Couples’, and ‘Perception of Social Relationships’. These encodings transformed the categorical data into a format that can be easily used by ML algorithms, thereby enhancing the comprehensiveness and depth of our analysis.

We evaluated several ML algorithms, including Artificial Neural Networks (ANNs), which are inspired by the structure and function of the human brain; K-Nearest Neighbors (KNNs), which makes predictions based on the similarity of data points; Random Forest (RF), which combines multiple decision trees to make robust predictions; RuleFit (RLF), which generates interpretable rules from the data; and Support Vector Machines (SVMs), which find optimal boundaries to separate different classes of data. These calculations were performed with the SIBILA tool [28]. Only models obtained with AUC > 0.75 were selected for later analysis with interpretability techniques. We employed techniques such as Feature Importance, which identifies the most influential predictors; Local Interpretable Model-Agnostic Explanations (LIME), which explains individual predictions by approximating the model locally; and Shapley Additive Explanations (SHAP), which assigns importance values to each feature based on their contribution to the model’s output. The employed workflow is depicted in Supplementary Figure S1.

These interpretability techniques enhance the transparency of our models by providing insights into the key predictive features and their contributions, making the decision-making process more accessible and understandable for practical applications.

## 3. Results

### 3.1. HSP Prevalence and Its Relationship with Participants’ Characteristics

From all participants, 689 individuals completed the evaluations, of whom 228 (33.1%) met the HSP criteria. This prevalence was higher in women, since 39.6% of women were HSP, while this percentage was only 11.3% in men. The chi-test revealed a significant association between sex and HSP criteria (χ^2^ = 44.925, *p <* 0.001). In fact, the r-HSP test score was significantly higher in women (14.5 ± 3.0 vs. 12.1 ± 3.3, *p* < 0.001; *d =* 3.097). In contrast, there were no differences in age regarding the HSP criteria (*p* = 0.985).

A one-way ANOVA was performed to evaluate the effect of nursing academic training on the HSP criteria; however, there was no statistically significant difference among the four academic years (*p* = 0.980). In contrast, those with previous academic formation, both university and technical, had a higher r-HSP score. In fact, HSP prevalence was higher in those with earlier university formation (42.9%) and technical (40.6%) than those without previous formation (30.6%) (*χ*^2^ = 6.236, *p* = 0.044).

The perception of the relationship with relatives had an important influence on the r-HSP score. This was reflected in a statistically significant lower score in those with a very unsatisfactory perception of the relationship with relatives (*p* < 0.001, *η*^2^ = 0.035). Interestingly, the score of those with an unsatisfactory perception of relations with relatives was slightly higher than those with highly satisfactory relations. This situation was mirrored with the relations with partners, since those with a very unsatisfactory perception showed the lower HSP score, although, in this time, the differences did not reach the significance level *(p* = 0.297), as well as with social relationships (*p* = 0.128).

### 3.2. The Diagnosis of HSP May Influence Emotional Intelligence and Communication Skills

Table 1 shows the emotional intelligence dimensions attending to the diagnosis or not of HSP. Our data show that emotional intelligence was independent of the HSP diagnosis, although the repair dimension was significantly higher in those individuals identified as HSP. Moreover, the HSP diagnosis showed an important effect on communication skills, since those with HSP had higher informative communication, empathy, respect, and global communication skills (Table 1).

### 3.3. Conflict Styles According to the HSP Diagnosis

**Figure 1:**
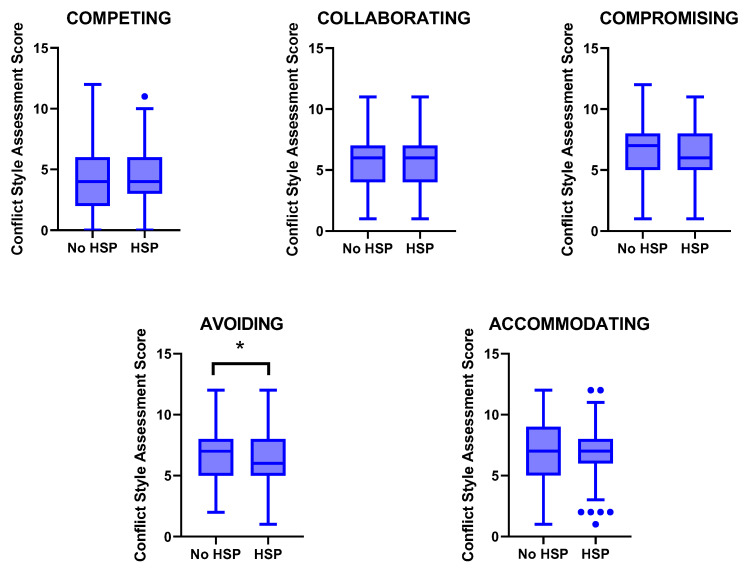
Boxplot of the different conflict styles according to the diagnosis of highly sensitive people (HSP). Statistical differences were evaluated by an independent *t*-test. * *p* < 0.050.

The individuals that took part in the study were characterized by accommodating as the main common conflict style, followed by compromising, avoiding, collaborating, and the least frequent, which was the competing style (Figure 1). When the different styles were compared attending to the HSP criteria, our data showed that HSP individuals were characterized by lower avoiding (*p* = 0.030, *d* = 2.101) and higher competing (*p* = 0.032, *d* = 2.647), although in this case, the differences might be a consequence of four HSP individuals with a high competing score (*p_corrected for outliers_* = 0.106) (Figure 1).

### 3.4. Prediction Models of the Presence of SPS Trait

To be able to predict the presence of the SPS trait in nursing students, a logistic regression model was developed using HSP diagnosis as a dependent variable. In this regard, we obtained a representative model that was able to explain 13.4% of the HSP score variability (*R*^2^ = 0.134, *p* < 0.001). Concretely, the variables that were finally included in the regression model were sex, previous health education, the perception of the relation with relatives, and informative communication (Table 2). From these variables, sex was the most relevant factor to predict the HSP diagnosis, since women showed a 5.95-fold increased risk of being diagnosed as HSP. Interestingly, the perception of relationships with family members was more important than social relationships or dating relationships to predict the HSP diagnosis.

Moreover, when the diagnostic performance was evaluated, our data revealed that the model was able to accurately diagnose 67.3% of HSP cases (Supplementary Table S1 shows the diagnostic parameters for the logistic regression model). Although we observed a high positive predictive value (the probability that the individual was HSP when the test result is positive), the performance of other indicators of diagnosis was moderate to low (Supplementary Table S1). This situation motivated the use of a machine learning (ML) framework to integrate features into a predictive model of HSP.

**Figure 2:**
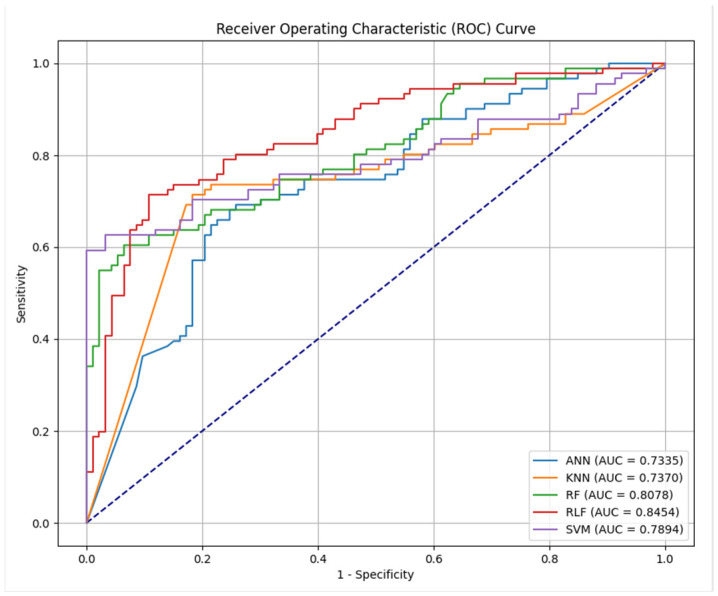
Receiver operating characteristic (ROC) curves for the ANN, KNN, RF, RLF, and SVM models. The AUC of each method is specified in the inset.

To evaluate the validity of all the ML algorithms in the test set (n = 184), the receiver operating characteristic curve (ROC) and AUC of the models are shown in Figure 2 (AUC  =  1 indicates perfect prediction; AUC  =  0.5 indicates random prediction). The other evaluation metrics, including accuracy, specificity, precision, and recall, for the models are presented in Supplementary Table S2. Overall, the findings proved that the RuleFit (RLF) model performed best among the prediction models, with an AUC value of 0.845 (Supplementary Table S2). The corresponding accuracy was 76.6%, the specificity was 74.2%, and the precision and recall were also over 75%. Therefore, the RLF model was selected as the best predictive model. The SVM algorithm also showed a high AUC, and several metrics showed a better yield than the RLF; however, the low recall (true positive rate) led us to choose the RLF algorithm.

**Figure 3:**
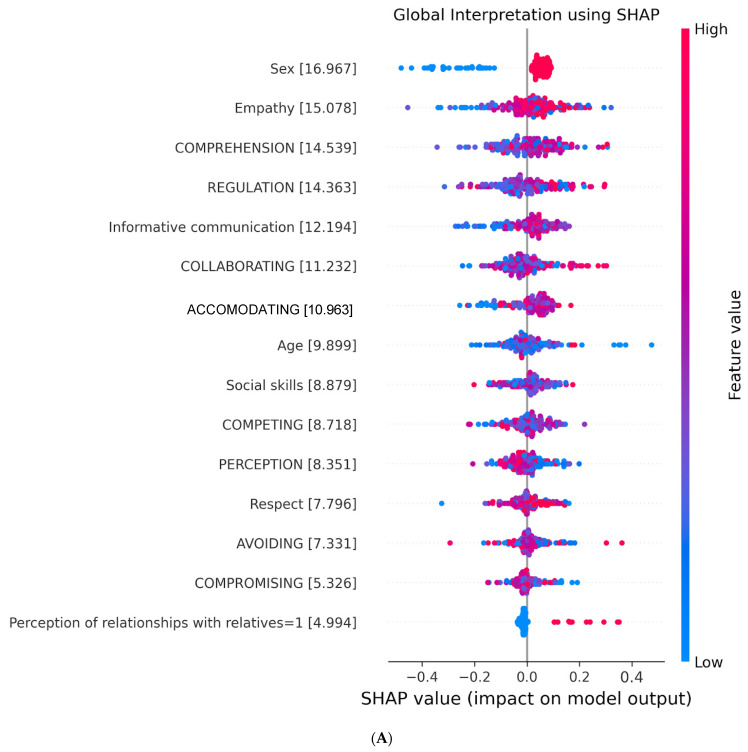

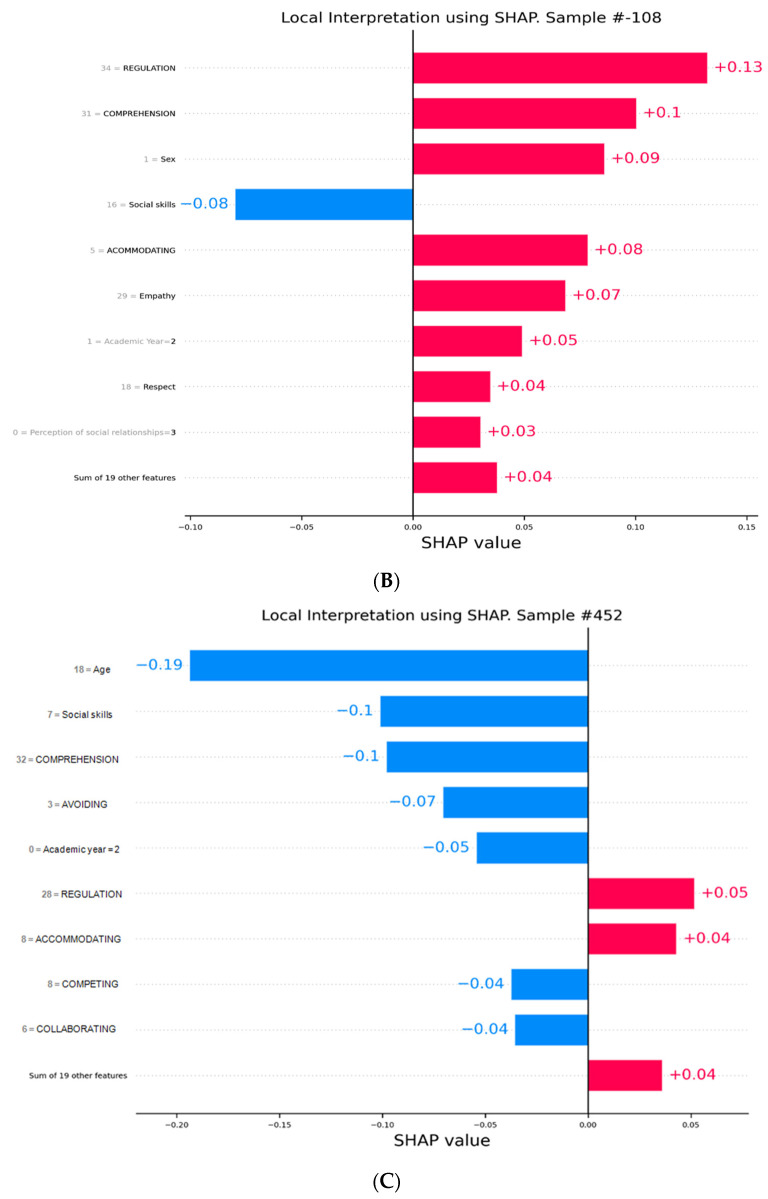
The impact of the input features on HSP predictions is visualized in (**A**), where each dot represents the effect of a feature on the prediction for one patient. The more intense the red color of the dots, the higher the value of the features, and the more intense the blue color of the dots, the lower the value of the features. Dots to the left of the *x*-axis represent patients with feature values decreasing the likelihood of an HSP prediction, and dots to the right indicate feature values increasing the likelihood of an HSP prediction. (**B**,**C**) show examples of individualized HSP prediction. (**B**) illustrates personalized risk factor analysis for a patient in the test set identified as HSP, while (**C**) demonstrates this analysis for an individual identified as No-HSP.

The contribution of each of the features in the RLF model is shown in Figure 3A. The features were ranked by their relative importance to HSP prediction according to the SHAP values of the model predictions. Sex was the most important feature of the prediction model, in the sense that women were identified as HSP. The next feature, in order of relevance, was empathy, with a higher HSP risk for those with high empathy, although several individuals with low empathy were also identified as HSP. In the ML model, comprehension and regulation, two dimensions of emotional intelligence, were identified as the third and fourth most notable features, respectively, while in the classic regression model, these characteristics were not present (Figure 3A). Based on the higher accuracy and sensibility of the ML model, we mapped the SHAP values and proposed a personalized risk factor analysis tool for explaining the HSP prediction for a particular individual, which is a scale from 0 to 1, visualizing the contribution of each feature to the prediction in probability. We showed the application of the personalized risk factor analysis method in one HSP (Figure 3B) and one participant not diagnosed as HSP (Figure 3C).

In addition to evaluating the predictive performance of the machine learning models, we also emphasized the interpretability of the selected model to gain insights into the factors driving its predictions. By employing techniques such as Feature Importance, Local Interpretable, and Shapley Additive Explanations (SHAP), we were able to identify the key predictive features and their contributions to the model’s decision-making process. This interpretability analysis enhances the transparency of our approach and provides valuable insights for practical applications, allowing stakeholders to understand the factors influencing the prediction of the SPS trait.

## 4. Discussion

The present work emerged with the aim of developing a predictive model capable of the early identification of those individuals with the sensory processing sensitivity (SPS) trait, who are identified as Highly Sensitive People (HSP). HSP are characterized by increased sensory processing and emotional reactivity. Understanding the prevalence and gender differences in HSP is essential for understanding individual variations in sensitivity and potential implications for their well-being and psychological functioning [13].

The prevalence data from the study revealed a global prevalence of 33%, remarkably similar to what was described in earlier studies [13,14], although there was a higher prevalence of HSP among women compared to men. However, no significant differences were found in age with respect to the HSP diagnosis. Previous studies have described that this trait is more frequent among nursing students and health professionals [29].

In the present work, the prevalence of HSP was similar throughout all the academic years of nursing students; however, those with previous university or technical health education showed a higher HSP prevalence than those without previous formation. Understanding how academic training can influence the manifestation of SPS is essential for a more complete understanding of this personality trait. The higher prevalence of HSP in those with previous university education and technical training was also described previously [30]. This demonstrates the possibility that exposure to broader and more specialized educational experiences may influence the development or recognition of high sensitivity, emphasizing the role of previous academic formation over the nursing academic training.

The perception of the relationship with relatives can influence the experience and manifestation of the characteristics of HSP [1]. Contrary to what may be expected, in the present work, those with a very unsatisfactory perception of relationships with relatives showed a higher probability of being HSP. Therefore, as indicated previously by Hofmann and Bitran, the perception of social relationships is relevant regarding the sensitivity and characteristics of HSP [31].

When exploring the relationship between HSP and emotional intelligence, it is important to consider the multifaceted construct that encompasses several components of emotional intelligence. HSP often exhibit heightened sensitivity to emotional cues, allowing them to be more attuned to their own emotions and those of others [32]. This enhanced emotional awareness may contribute to their ability to recognize and accurately identify emotions in themselves and others. HSP may have a greater capacity for perceiving and understanding subtle emotional expressions and nonverbal cues [4]. In this line, we observed higher emotional repair in HSP, which may be a consequence of the presence of the SPS trait in these individuals.

The association between HSP and communication skills is also of great relevance. Firstly, it is important to note that there are individual differences among HSP, and not all HSP will have the same level of communication abilities. HSP often have heightened sensory processing, making them more susceptible to sensory overload in certain environments or during intense communication situations [26]. This sensory overload can potentially impact their ability to effectively communicate and process information in real time. HSP may require more time and space to regulate their sensory input, which could affect their communication style [1].

Concretely, and in line with the current observations, HSP often show a heightened ability for empathy. They may have an innate ability to understand and share the emotions of others, leading to greater compassion and interpersonal sensitivity [32]. This empathetic nature allows HSP to connect with others on a deeper level and respond empathically to their emotional experiences. Similarly, respect and informative communication were also more elevated in HSP, which may also be associated with the characteristics of HSP. For instance, they are characterized by heightened awareness that can lead to a more respectful and considerate approach in interpersonal relationships. Moreover, highly sensitive individuals often engage in introspection and self-reflection. This self-awareness can lead to a better understanding of their own actions and how they impact others, fostering a greater sense of respect in their interactions.

In the context of conflict styles, the Thomas–Kilmann Conflict Mode Instrument (TKI) is a widely used assessment that measures individuals’ preferred approaches to conflict resolution. Attending to the data obtained, HSP showed lower avoiding and compromising styles compared with those without the SPS trait. These observations are somewhat controversial because, while the relationship between HSP and conflict styles has not been extensively researched, it is possible to discuss potential conflict styles that HSP may show based on general observations and characteristics associated with high sensitivity. For instance, HSP typically have a strong aversion to conflict and a desire for harmony [30]. This preference may lead them to avoid or downplay confrontational or challenging communication situations. HSP may prioritize keeping peace and avoiding arguments, which can sometimes result in less assertive or direct communication styles, potentially hindering their ability to effectively express themselves in certain contexts.

Therefore, the observation that HSP are less avoidant is, in principle, contrary to what one would expect. On the other hand, HSP often engage in the deep processing of information and emotions. They may take more time to carefully consider their thoughts and responses before communicating them [26]. While this trait can contribute to thoughtful and well-considered communication, it may also result in slower response times during fast-paced or spontaneous conversations, which can be perceived as a lack of communication skills in certain situations. This could partially explain the lower compromising, a conflict resolution style that aims to find a mutually acceptable solution to the conflict while maintaining some assertiveness and cooperativeness, observed in the data derived from HSP. However, as commented above, it is important to note that individual differences exist within the HSP population, and not all HSP will have the same conflict style [33]. In addition, an HSP’s preferred conflict style can also be influenced by factors such as cultural background, individual personality traits, and contextual factors [17].

Focusing on the main aim, namely, to find a predictive model to detect HSP early, a double approach has been conducted. Firstly, a classic logistic model was developed using HSP diagnosis as the dependent variable. This procedure revealed that sex, previous health education, and the perception of relationships with relatives were the main predictors of HPS, over emotional intelligence and conflict resolution styles. Previously, Drndarevic et al. have described the link between SPS and emotional intelligence [34], which reinforces, in part, our observations. Moreover, informative communication, a dimension of communication skills, also predicted HSP but with less relevance than the previous factors. However, although this model yielded a high positive predictive value, other indicators like specificity were low, which encouraged the use of a machine learning (ML) framework to integrate features into a predictive model of HSP.

Attending to the ML model, as with the previous logistic one, sex was the main predictor feature for identifying HSP. However, in this case, earlier health training and the perception of relationships were not as relevant as in the classic model. Considering the higher values obtained regarding sensitivity metrics in the ML model, this procedure would be more suitable to predict the presence of the SPS trait, and therefore, the characteristics shown by this model should be considered to a greater extent than in the classic model. Other data that support this idea come from the relevance of empathy in the ML model. In this case, empathy was, after sex, the most significant factor. Additionally, McCarthy et al. also noted the importance of evaluating emotional intelligence dimensions, such as empathy, to find HSP [35].

Communication skills, specifically informative communication, were also important predictors in both models. There are several issues that could be related to this fact. HSP often engage in the deep processing of information and emotions [36]. They may take more time to carefully consider their thoughts and responses before communicating them. While this trait can contribute to thoughtful and well-considered communication, it may also result in slower response times during fast-paced or spontaneous conversations, which can be perceived as a lack of communication skills in certain situations. Moreover, HSP are often highly attuned to nonverbal cues and subtle emotional expressions [13]. This sensitivity can be an asset in communication, as they may notice nuanced emotions and underlying messages. However, it can also make them more sensitive to nonverbal cues that may be perceived as negative or critical, potentially leading to heightened emotional reactions or withdrawal in communication. Additionally, effective communication skills are influenced by a combination of factors including personality traits, individual experiences, and environmental factors [23].

When we consider conflict resolution styles as HSP predictors, the most important styles were collaborating and accommodating, although in this case, the trend was not as clear as with the other features. However, in the accommodative style, it does seem that the higher the score, the greater the probability of being HSP, which is in line with the previous observations of Labrague [37].

Overall, the transparency and interpretability of our machine learning approach to detect SPS have significant implications for educational settings. By understanding the factors driving the prediction of the SPS trait, educators can develop targeted interventions and support strategies for HSP students. The interpretability techniques employed in our study, such as SHAP, provide actionable insights into the key predictive features and their contributions to the model’s decision-making process. This enhanced transparency enables educators to tailor their approaches based on the specific needs and characteristics of individual students, ultimately promoting a more inclusive and supportive learning environment for HSP individuals.

Several limitations should be considered when interpreting the findings of this study. Firstly, the cross-sectional nature of our research design limits the ability to establish causal relationships between the observed variables and the presence of the SPS trait. Future longitudinal studies are needed to confirm the predictive value of the identified factors and to assess the stability of the SPS trait over time. We also thought that future work would receive help from the inclusion of a desirable prediction measure to determine the role of specific individual characteristics in the HSP diagnosis, as in the present work. It would be of scientific interest to ascertain the temporal dynamics of individual factors figuring out the components of SPS associated with heightened responsiveness in this trait, but that manifest distinct trajectories depending on individual variations [3].

Secondly, our sample consisted of nursing students from a single institution, which may limit the generalizability of the results to other populations or settings. Further validation of our findings in diverse samples, including practicing nurses and individuals from different cultural backgrounds, is necessary to establish the external validity of the predictive model. Lastly, while we employed a comprehensive set of variables in our analysis, there may be additional factors, such as genetic predispositions or early life experiences, that could influence the development and expression of the SPS trait. Future research should consider incorporating a broader range of variables to enhance the predictive power of the model and provide a more comprehensive understanding of the SPS trait.

## 5. Conclusions

The findings of this study supply valuable information on the prevalence and gender differences in HSP. The significantly higher prevalence of HSP among women suggests that women may be more likely to have the SPS trait than men. Additionally, women showed higher sensitivity scores, indicating a more intense experience of sensory processing and emotional reactivity. HSP demonstrated greater empathy and decision-making skills, allowing them to understand and connect with the emotions of others. This empathic nature can contribute to self-awareness and emotional regulation. Therefore, the early detection of HSP may help them to better regulate and manage their feelings. Several prediction models have been developed, and it can be concluded that sex, empathy, and communication skills are the main factors when predicting the diagnosis of HSP. Previous health education and the perception of relationships with relatives may also be important.

Nevertheless, when considering HSP prediction, it is important to note that while some HSP may have certain qualities that can contribute to effective emotion regulation, not all HSP will have the same level of competence. Therefore, an individualized machine learning predictive model, as conducted in the present work, may be of better interest to identify HSP early, since the relevance of the different prediction features may differ depending on factors such as cultural or environmental and other psychological variables.

In addition to the aforementioned, it is important to consider the implications of these findings in various contexts. Exploring this relatively understudied personality trait contributes to a more comprehensive understanding of human behavior and individual differences. By recognizing and acknowledging the prevalence of high sensitivity, researchers, practitioners, and society at large can develop more inclusive and tailored approaches in various domains, including mental health, education, and workplace environments.

Further investigation is called for to expand upon these findings, employing larger and more diverse samples to provide a broader representation of the population. Additionally, longitudinal studies could help assess the stability of high sensitivity over time and examine its potential associations with various outcomes, such as well-being, interpersonal relationships, and career choices.

## Figures and Tables

**Table 1:** Emotional intelligence (as measured by the TMMS24 test) and communication skills (evaluated by the EHC-PS test) attending to the diagnosis or not of HSP.

	No HSP (n = 461)	HSP (n = 228)	*p* (*t*-Test)
Attention	27 ± 6	27 ± 7	0.687
Clarity	23 ± 7	24 ± 7	0.462
Repair	24 ± 7	26 ± 7	0.033
Total TMMS24	75 ± 14	76 ± 15	0.269
Informative communication	30.0 ± 3.4	30.7 ± 3.4	0.011
Empathy	25.8 ± 3.5	26.4 ± 3.2	0.030
Respect	16.1 ± 1.8	16.4 ± 1.8	0.038
Social skill	15.5 ± 3.5	15.4 ± 3.2	0.840
Total EHC-PS	87.4 ± 9.2	88.9 ± 8.9	0.036

Data represent mean ± sd. Significant differences were evaluated by the *t*-test. Effect size (Cohen’s d) for repair dimension was *d* = 6.516; informative communication *d* = 9.117; empathy *d* = 3.394; total EHC-PS = 3.417.

**Table 2:** Multivariable logistic regression model, using HSP diagnosis as dependent variable. The table shows only those features with an odd ratio associated with a statistically significant effect.

	B Coeff	S.E	Wald	Sig	OR (CI 95%)
Sex (women)	−1.786	0.283	39.889	<0.001	5.952 (3.425–10.417)
Previous health education	0.288	0.107	7.299	0.007	1.334 (1.082–1.645)
Perception of relationship with relatives	−0.231	0.134	2.965	0.087	1.261 (0.968–1.639)
Informative communication	0.063	0.026	5.895	0.015	1.065 (1.012–1.120)

B: standard coefficient. S.E: standard error. Sig: associated *p*-value. OR: odd ratio. CI: confidence interval.

## Data Availability

The data that support the findings of this study are openly available in the Mendeley Data Repository at http://doi.org/10.17632/279pxc72hy.1, accessed on 1 February 2024. The data can be accessed at https://data.mendeley.com/datasets/279pxc72hy/1, accessed on 1 February 2024. The code for the machine learning (ML) procedure and other ML data can be accessed under reasonable request to Dr. Juan José Hernández Morante (jjhernandez@ucam.edu).

## Supplementary Materials

The following supporting information can be downloaded at: https://www.mdpi.com/article/10.3390/ejihpe14040059/s1; Figure S1: schematic representation of the machine learning workflow employed in the study, Table S1: diagnostic test parameters comparing the effectiveness of the logistic regression model versus the HSP diagnostic test (used as gold-standard), Table S2: diagnostic test parameters comparing the effectiveness of the different machine learning algorithms employed in the present study. Those algorithms with AUC values < 0.60 were excluded (DT, RP, and XGBOOST).
