# Supplementary material for: An Interpretable Machine Learning Approach to Predict Sensory Processing Sensitivity Trait in Nursing Students
Source: Eur J Investig Health Psychol Educ. 2024 Apr 2;14(4):913–28. doi: 10.3390/ejihpe14040059 (PMC11049261; doi:10.3390/ejihpe14040059)

SUPPLEMENTARY MATERIAL

Supplementary Table S1. Diagnostic test parameters comparing the effectiveness of the logistic regression model versus de HSP diagnostic test (used as gold-standard).

		**Predicted**		**Total**
		No HSP	HSP	
Observed	No HSP	427	31	93,2
	HSP	191	30	13,6
Total				67,3

Sensitivity: 69% (65-73%); Specificity 49% (37-62%), PPV^+: 93% (91-96%); PPV^-: 14% (9-18%).

Supplementary Table S2. Diagnostic test parameteres comparing the effectiveness of the different machine learning algorithms employed in the present study. Those algorithms with AUC values < 0.60 were excluded (DT, RP and XGBOOST).

	**ANN**	**KNN**	**RF**	**RLF**	**SVM**
Accuracy	0.6793	0.6359	0.7011	0.7663	0.7717
Precision	0.6538	0.6034	0.7045	0.75	0.8769
F1	0.6974	0.6763	0.6927	0.7701	0.7308
Recall	0.7473	0.7692	0.6813	0.7912	0.6264
Specificity	0.6129	0.5054	0.7204	0.7419	0.914
AUC-ROC	0.7335	0.737	0.8078	0.8454	0.7894

Supplementary Figure S1: Schematic representation of the machine learning workflow employed in the study. The collected data underwent preprocessing steps including outlier removal, variance analysis, one-hot encoding of categorical variables, and correlation analysis. The preprocessed data was then used to train multiple machine learning models, namely Artificial Neural Network (ANN), K-Nearest Neighbors (KNN), Random Forest (RF), RuleFit (RLF), and Support Vector Machine (SVM), using the SIBILA tool. The trained models were evaluated based on their performance metrics, and the best-performing model was selected for further analysis. Interpretability techniques such as Feature Importance, and Shapley Additive Explanations (SHAP) were applied to the selected model to gain insights into the predictive features and their contributions to the model's predictions.

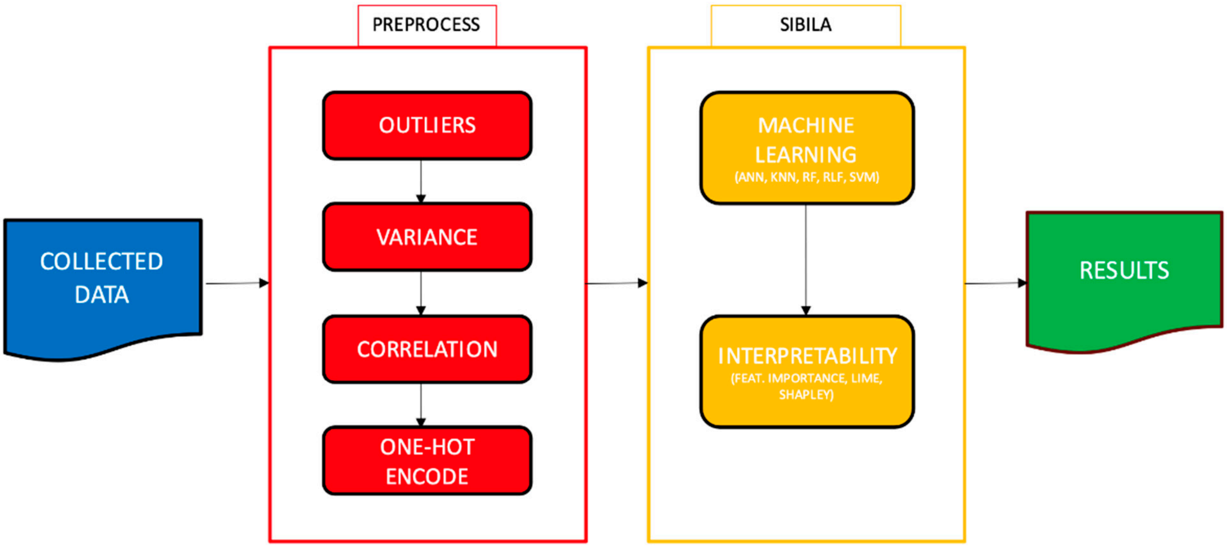

Supplement: Supplementary file 1 [file ejihpe-14-00059-s001.zip › ejihpe-2878626-supplementary.pdf]
